# Taxifolin targets HMGCR, LDLR and APOB to ameliorate hyperlipidemia: insights from systems pharmacology and nanoemulsion-based delivery

**DOI:** 10.1515/biol-2025-1392

**Published:** 2026-10-22

**Authors:** Abdullah R. Alanzi, Iman Fatima, Muhammad Umer Ashraf, Muhammad Nasir Hayat Malik, Rashed N. Herqash, Mostafa E. Abugrain, Muhammad Atif, Munawar Abbas, Gideon F.B. Solre

**Affiliations:** Department of Pharmacognosy, College of Pharmacy, King Saud University, 11451 Riyadh, Saudi Arabia; Faculty of Pharmacy, The University of Lahore, Lahore, Pakistan; Center for Research on Medicinal, Aromatic, and Poisonous Plants, DSR, King Saud University, P.O. Box 2457, Riyadh, Saudi Arabia; Department of Pharmacognosy, Faculty of Pharmacy, University of Tripoli, Tripoli, Libya; College of Food Science and Technology, Henan University of Technology, Zhengzhou, Henan, 450001, China; Department of Chemistry, Thomas J. R. Faulkner College of Science and Technology, University of Liberia, Monrovia, Montserrado County, Liberia

**Keywords:** taxifolin, hyperlipidemia, HMGCR, APOB, LDLR, TXF-NE

## Abstract

Hyperlipidemia is a major risk factor for cardiovascular disease, highlighting the need for safer and effective therapeutic options. This study evaluated the anti-hyperlipidemic potential of taxifolin (TXF) and its nanoemulsion formulation (TXF-NE) using integrated computational and experimental approaches. Network pharmacology identified 177 common targets between TXF and hyperlipidemia, with enrichment analyses linking these targets to lipid metabolism, cholesterol regulation, and inflammatory pathways. Protein-protein interaction analysis revealed key lipid-regulating genes, including HMGCR as a major hub target. Molecular docking predicted favorable interactions of TXF with several lipid metabolism related proteins, including HMGCR, LDLR, APOB, APOA1, and ACSM2A. The predicted interactions provide structural hypotheses regarding potential TXF protein associations. *In-vivo*, TXF significantly improved serum lipid profiles and liver function enzymes. Gene expression and protein analyses exhibited downregulation of HMGCR and APOB along with upregulation of LDLR, indicating suppression of cholesterol synthesis, reduced lipoprotein production, and enhanced LDL clearance. Notably, TXF-NE exhibited high drug content and better therapeutic efficacy relative to free TXF, as reflected by greater improvements in lipid parameters. Overall, these findings demonstrate that TXF alleviates hyperlipidemia through a multi-target mechanism, while nanoemulsion-based delivery enhances its therapeutic potential for managing hyperlipidemia and associated hepatic dysfunction.

## 1 Introduction

Hyperlipidemia is a multifactorial metabolic disorder intricately linked with the development and progression of atherosclerotic cardiovascular diseases (CVDs), which continue to be the leading cause of global mortality. The condition is typified by elevated serum concentrations of total cholesterol (TC), triglycerides (TG), low-density lipoprotein (LDL), and very low-density lipoprotein (VLDL), along with reduced levels of high-density lipoprotein (HDL) [1], 2]. This dysregulation in lipid metabolism compromises vascular homeostasis, induces endothelial dysfunction, and facilitates the formation of lipid-rich atherosclerotic plaques, ultimately increasing the risk of coronary artery disease, myocardial infarction, and ischemic stroke. Importantly, the pathophysiology of hyperlipidemia is complex, involving interplay of genetic predispositions, hormonal imbalances, lifestyle factors, and comorbidities such as diabetes mellitus [3], [4], [5], [6], [7].

From a molecular standpoint, dyslipidemia results from aberrations in the synthesis, transport, and clearance of lipoproteins. Enzymes and proteins such as HMG-CoA reductase (HMGCR), apolipoprotein B (APOB), proprotein convertase subtilisin/kexin type 9 (PCSK9), LDL receptors (LDLR), and peroxisome proliferator-activated receptors (PPARs) orchestrate the regulation of lipid homeostasis. Mutations or dysregulation in these molecules can lead to increased endogenous cholesterol synthesis, impaired LDL clearance, and elevated atherogenic lipoproteins in circulation. Moreover, hepatic overproduction of VLDL and defective lipoprotein lipase activity can aggravate TG accumulation, particularly in insulin-resistant and diabetic states. Accumulating evidence also highlights the role of oxidative stress and chronic inflammation in exacerbating lipoprotein oxidation and vascular injury, further accelerating atherogenesis [8], [9], [10], [11], [12], [13], [14].

The pharmacological management of hyperlipidemia has traditionally centered on statins, which inhibit HMGCR to reduce LDL-cholesterol levels. Despite their proven efficacy, statins are associated with adverse effects such as myopathy, hepatotoxicity, and insulin resistance, leading to poor compliance in some patients. Furthermore, a substantial proportion of individuals with high cardiovascular risk do not achieve target lipid levels even with high-dose statins, underscoring the issue of residual risk. Other pharmacotherapies, including ezetimibe, fibrates, niacin, and bile acid sequestrants, offer modest additional benefits but are often limited by adverse events or drug interactions. Although recent advances such as PCSK9 inhibitors have demonstrated superior lipid-lowering effects, their high cost and limited accessibility hinder widespread clinical application [12], [13], [14], [15], [16], [17], [18]. This highlights an unmet need for alternative or adjunctive therapies that are not only effective but also well tolerated.

In recent years, attention has turned towards plant-derived bioactive compounds for the management of metabolic disorders. Among them, taxifolin (TXF), a naturally occurring flavanonol found in onions, grapes, milk thistle, larch trees, and other plant sources, has emerged as a promising candidate due to its broad pharmacological spectrum. TXF exhibits potent antioxidant, anti-inflammatory, and hepatoprotective effects, and has shown favorable outcomes in experimental models of oxidative stress, liver injury, and metabolic syndrome [19], [20], [21]. More relevantly, emerging data suggest that TXF maintains rat’s normal lipid profiles in their serum and liver by promoting lipid excretion [22]. Animals treated with TXF have also shown decreased serum and liver levels of thiobarbituric acid reactive substances [23]. These multifaceted actions position TXF as a unique therapeutic agent that addresses both the lipid-centric and inflammatory aspects of hyperlipidemia.

Considering the limitations of current lipid-lowering therapies and the rising interest in phytochemicals as complementary therapeutic agents, elucidating the role of TXF in lipid regulation represents a relevant and timely research direction. The present study aims to comprehensively evaluate the potential of TXF in the management of hyperlipidemia by elucidating its impact on key molecular markers, lipid profile parameters, and mechanistic pathways. Recent advances in computational biology and bioinformatics have expanded the application of computational approaches for investigating protein functions, molecular interactions and regulatory mechanisms. These developments highlight the growing utility of computational methods for identifying biologically relevant molecular features and regulatory interactions [24], [25], [26], [27]. In this context, the present study integrates network pharmacology, protein-protein interaction (PPI) analysis, functional enrichment, and molecular docking with experimental validation to investigate the potential molecular mechanisms underlying the antihyperlipidemic effects of TXF.

## 2 Materials and methods

### 2.1 Network pharmacological analysis

The chemical structures of TXF were retrieved in both SMILES and SDF formats from PubChem [28]. The structural information was processed using three distinct target prediction platforms: PharmMapper [29], SuperPred [30], and WAY2DRUG [31]. The resulting protein targets were standardized and consolidated via the UniProt [32]. To identify disease targets, an extensive search was conducted using the term “Hyperlipidemia” across GeneCards [33], DisGeNET [34], and the Comparative Toxicogenomics Database (CTD) [35]. To refine the specificity of the targets, results from GeneCards were filtered based on median relevance scores. The overlap between the TXF-related targets and hyperlipidemia-associated genes was visualized using Venny 2.1 software, allowing us to identify potential therapeutic targets linked to the anti-hyperlipidemic effects of TXF [36].

#### 2.1.1 Construction of PPI network

To explore the interaction between TXF and hyperlipidemia-related targets, a PPI network was constructed using the STRING database, specifically focusing on human proteins [37]. The network was visualized and analyzed through Cytoscape software. Key components within the network were identified using the cytoHubba plugin’s degree ranking method, which highlighted the top 50 hub genes based on their connectivity [38].

#### 2.1.2 Functional enrichment analysis of hub genes

The identified hub genes were subjected to functional enrichment analysis using the ShinyGO platform, which facilitates the exploration of Gene Ontology (GO) and Kyoto Encyclopedia of Genes and Genomes (KEGG) pathways. This analysis aimed to elucidate the key biological processes and pathways associated with the hub genes. A significance threshold of *p* < 0.05 was applied to identify the 20 most relevant GO terms, encompassing biological processes (BP), cellular components (CC), and molecular functions (MF), alongside the top 20 significant KEGG pathways [39], 40].

### 2.2 Molecular docking protocol

#### 2.2.1 Preparation of protein structures

The 3D crystal structures of five pivotal proteins APOA1 (PDB ID: 1AV1), HMGCR (PDB ID: 1HW8), LDLR (PDB ID: 1N7D), CoA-Synthase acyl-CoA synthetase medium-chain family member 2A (ACSM2A; PDB ID: 2VZE), and APOB100 (PDB ID: 9EA7) were obtained from the RCSB Protein Data Bank [41]. Using MOE software, these proteins were prepared by systematically removing non-essential elements such as solvent molecules, cofactors, and redundant chains. Hydrogen atoms were added, and protonation states were adjusted to reflect physiological pH (7.4). Energy minimization was performed utilizing the AMBER10 forcefield, with a convergence threshold set at 0.1 kcal/mol/Å [42].

#### 2.2.2 Ligand preparation and optimization

The molecular structures of TXF and SIM were sourced from PubChem in SMILES format. Using MOE’s molecular modeling tools, these 2D structures were converted into optimized 3D configurations. Energy minimization was conducted with the MMFF94x force field, applying a convergence threshold of 0.01 kcal/mol. Atomic partial charges were assigned according to the AMBER charge model. Comprehensive conformational sampling was employed to identify the lowest-energy conformers, which were subsequently used for molecular docking studies [43].

#### 2.2.3 Docking configuration

Molecular docking studies were performed using the integrated docking platform in MOE. Since suitable experimentally defined co-crystallized ligand poses were not consistently available for all selected protein structures, conventional native-ligand redocking validation could not be uniformly performed across the selected targets. Therefore, potential ligand-binding regions were identified using the Site Finder module of MOE, which predicts candidate binding cavities based on the geometric and physicochemical characteristics of the protein surface. The predicted binding sites were subsequently used for docking of TXF and SIM. Docking simulations were performed using the Triangle Matcher algorithm for ligand placement, followed by refinement using the Induced Fit protocol. Initial pose evaluation was performed using the London dG scoring function, followed by rescoring using the GBVI/WSA dG scoring function. Multiple docking trials were performed for each ligand protein system to explore alternative binding orientations and conformations. The resulting poses were examined based on predicted docking scores and protein ligand interactions, including hydrogen bonding and hydrophobic contacts. Since native ligand redocking was not performed, the docking results were considered predictive and hypothesis-generating rather than formally validated predictions of experimental binding affinity or biological activity [44], 45].

### 2.3 Chemicals and reagents

Triton WR-1339 (TWR-1339), triton X-100, TXF, simvastatin (SIM), dimethyl sulfoxide (DMSO), trizol, ethanol were purchased from Sigma Aldrich, Germany, peppermint oil from Standard Manufacturing Company Ltd., Pakistan, PEG 400, tween 80 from VWR Chemicals, USA, Wiz Script cDNA Synthesis Kit (High Capacity) from Wiz Bio Solutions, USA, SYBR Green qPCR mix from Zokeyo, China.

### 2.4 Animals

Male Sprague-Dawley (SD) rats (6–8 weeks old, 170–200 g) were purchased from the University of Veterinary and Animal Sciences, Lahore, Pakistan, and housed in the animal care facility of the Faculty of Pharmacy, University of Lahore under standard conditions. They were given a week to get used to their surroundings. The care given to the animals complied with OECD norms [46]. The Institutional Research Ethics Committee, Faculty of Pharmacy, University of Lahore, authorized the experimental protocol [47]. Two independent *in-vivo* experiments were conducted using separate cohorts of male SD rats. Animals used in the first experiment were not reused in the second experiment. In each experiment, animals were randomly assigned to five groups, with six animals per group (*n* = 6). Therefore, a total of 60 animals were used across the two *in-vivo* experiments. Random assignment to experimental groups was performed before treatment initiation. All animals completed their respective experimental protocols and were included in the final analyses. No animals were excluded. A formal *a priori* power or sample-size calculation was not performed, and this is acknowledged as a limitation of the study. Formal allocation concealment and blinding of outcome assessment were not implemented in the original experiments and have not been claimed in the present study.

**Ethical approval:** The research related to animal use has been complied with all the relevant national regulations and institutional policies for the care and use of animals, and has been approved by the Institutional Research Ethics Committee, Faculty of Pharmacy, University of Lahore (approval number: IREC-2024-19).

### 2.5 Hyperlipidemia induction by TWR-1339 followed by triton X-100

Hyperlipidemia was induced by a single oral dose of TWR-1339 (400 mg/kg) in rats. Animals were subsequently daily treated (orally) with triton X-100 (50 mg/kg) followed by either SIM (20 mg/kg) or various doses of TXF (25 and 50 mg/kg), 72 h post-induction, for one week. Upon completion of the study period, animals were euthanized with pentobarbital sodium (200 mg/kg) and killing was ensured by cervical dislocation. Care was taken to minimize the suffering of the animals throughout the study. A well-trained laboratory technician performed all euthanasia procedures in accordance with established standard protocols. Blood samples were collected via cardiac puncture in serum-separator (gel) tubes and subsequently analyzed for lipid profile and LFT analyses. Liver samples were collected in trizol reagent for real-time (RT) polymerase chain reaction (PCR) [48], 49].

#### 2.5.1 Study design

Animals were divided into five groups with six animals in each group (*n* = 6):

1. Normal control: No treatment.
2. TWR-1339 (400 mg/kg): TWR-1339 single dose followed by triton X-100 for one week.
3. SIM (20 mg/kg): TWR-1339 single dose followed by triton X-100 and SIM for one week.
4. TXF (25 mg/kg): TWR-1339 single dose followed by triton X-100 and TXF (25 mg/kg) for one week.
5. TXF (50 mg/kg): TWR-1339 single dose followed by triton X-100 and TXF (50 mg/kg) for one week.

#### 2.5.2 Lipid profile and LFT analyses

At the end of the experiment, animals were sacrificed. Before their decapitation, the rats were sedated, and their blood was drawn into sterile, serum-separator (gel) tubes. Following a 10 min centrifugation, the serum samples were extracted for biochemical analysis. Serum TC, TG, LDL, HDL and VLDL were measured directly using standard kit. Serum AST, ALT and ALP activities were measured by ELISA using MyBioSource kits (MBS165203, MBS269614, MBS264975) [50].

#### 2.5.3 RT-qPCR

Total RNA was extracted from liver tissue using the TRIzol™ reagent (Invitrogen, USA), following the manufacturer’s instructions. The concentration and purity of the isolated RNA were determined using a NanoDrop spectrophotometer (Thermo Scientific, USA). Complementary DNA (cDNA) was synthesized from 1 μg of total RNA using the WizScript™ cDNA Synthesis Kit according to the manufacturer’s protocol. The resulting cDNA was diluted and used as the template for qPCR amplification. RT-qPCR was performed using Zokeyo 2× SYBR Green qPCR Master Mix on an Applied Biosystems StepOne™ RT PCR System (Thermo Fisher Scientific, USA). The relative expression levels of target genes were calculated using the ΔΔCT method. HPRT was used as the internal reference gene for normalization. Primer sequences used for each gene are listed in Table S1 (Figure 1) [51].

**Figure 1:**
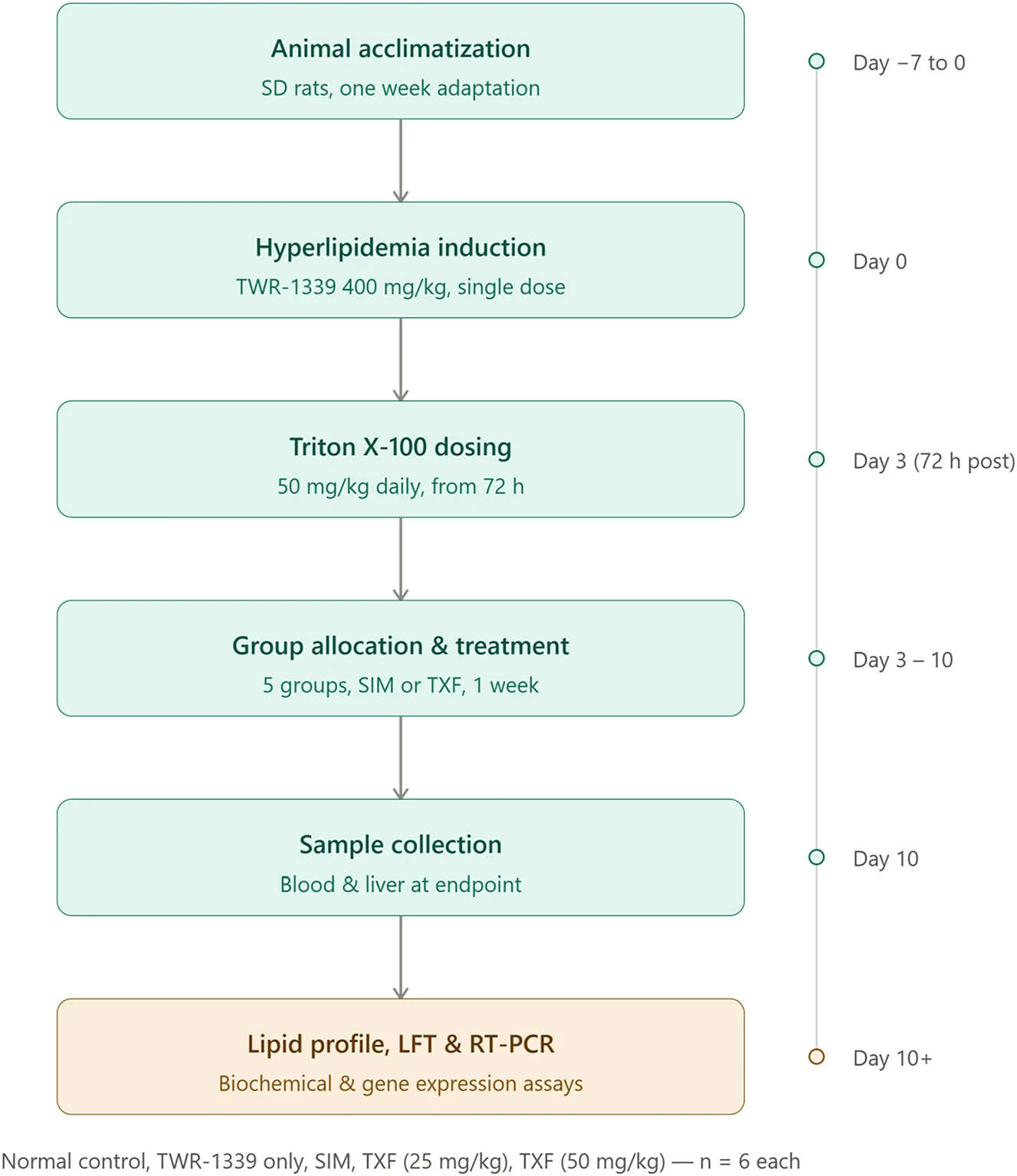
Flowchart of anti-hyperlipidemic evaluation of TXF.

### 2.6 Preparation of TXF-nanoemulsion (TXF-NE)

The water titration approach was used to create a TXF-NE formulation by adjusting the proportions of water, oil, surfactant, and cosurfactant while maintaining the TXF concentration constant in each instance. The drug was mixed in an accurate quantity of oil (peppermint oil) while keeping on gentle stirring on a hot plate magnetic stirrer. To this, surfactant (tween 80) and cosurfactant (PEG 400) were added and gently mixed for 10 min at room temperature on a high-speed overhead stirrer. Water was then gradually added until a transparent or clear NE was obtained. The stirring speed was kept at 1,500 rpm for all the formulations and the stirring time was 30 min. The mean zeta size, polydispersity index and zeta potential of the optimized formulations were subsequently measured (Table 1) [52].

**Table 1:** Composition of TXF-NE.

Ingredients	Quantity
TXF	1,000 mg
Peppermint oil	10 ml
Tween 80	10 ml
PEG 400	10 ml
Distilled water	20 ml (Q.S)

#### 2.6.1 Characterization and evaluation of TXF-NE

##### 2.6.1.1 Visual inspection

The NE was visually assessed for consistency, optical clarity, and fluidity.

##### 2.6.1.2 Zeta size and potential measurement

A zeta-sizer nano series-ZS (Malvern Instruments, Malvern, UK) was utilized to evaluate the TXF-NE zeta potential, and particle size at 25 °C. To assess particle size and size distribution, the dynamic light scattering (DLS) technique was employed. The zeta-potential (mV) of the NE was measured using the same device in Laser Doppler Velocimetry (LDV) mode [53], 54].

##### 2.6.1.3 Drug content determination in TXF-NE

The assay of drug content in NE-formulations was conducted on a UV spectrophotometer at *λ*_max_ of 289 nm. The NE (5 ml) was centrifuged at 4,000 rpm with 5 ml of ethanol for 20 min at RT. 3 ml of supernatants was collected and after suitable dilution with ethanol, they were assayed for drug content. The absorbance of the sample was compared with the absorbance of the standard TXF solution, which was prepared by dissolving 5 mg of TXF 5 ml ethanol, then 3 ml of this solution was taken and diluted up to 10 ml with ethanol [52].

$$\text{Drug loading }\left(100\;\%\right)=\frac{\text{Absorbance}\;\text{of}\;\text{sample}}{\text{Absorbance}\;\text{of}\;\text{standard}}\times 100$$

##### 2.6.1.4 Fourier-transform infrared spectroscopy (FTIR)

FTIR spectroscopy of TXF, tween 80, PEG 400, and the drug-loaded emulsion were performed to confirm drug loading into the NE. The analysis was performed using IR prestige-21 (Shimadzu, Japan). Using liquid assembly, the transmittance mode was used to record the spectra based on the wavenumber (4,000–500 cm^−1^) [55], [56], [57].

### 2.7 Antihyperlipidemic assessment of TXF-NE

Anti-hyperlipidemic effects of TXF-NE were measured according to the above mentioned methods (Figure 2). Study design of the experiment is as follows:

**Figure 2:**
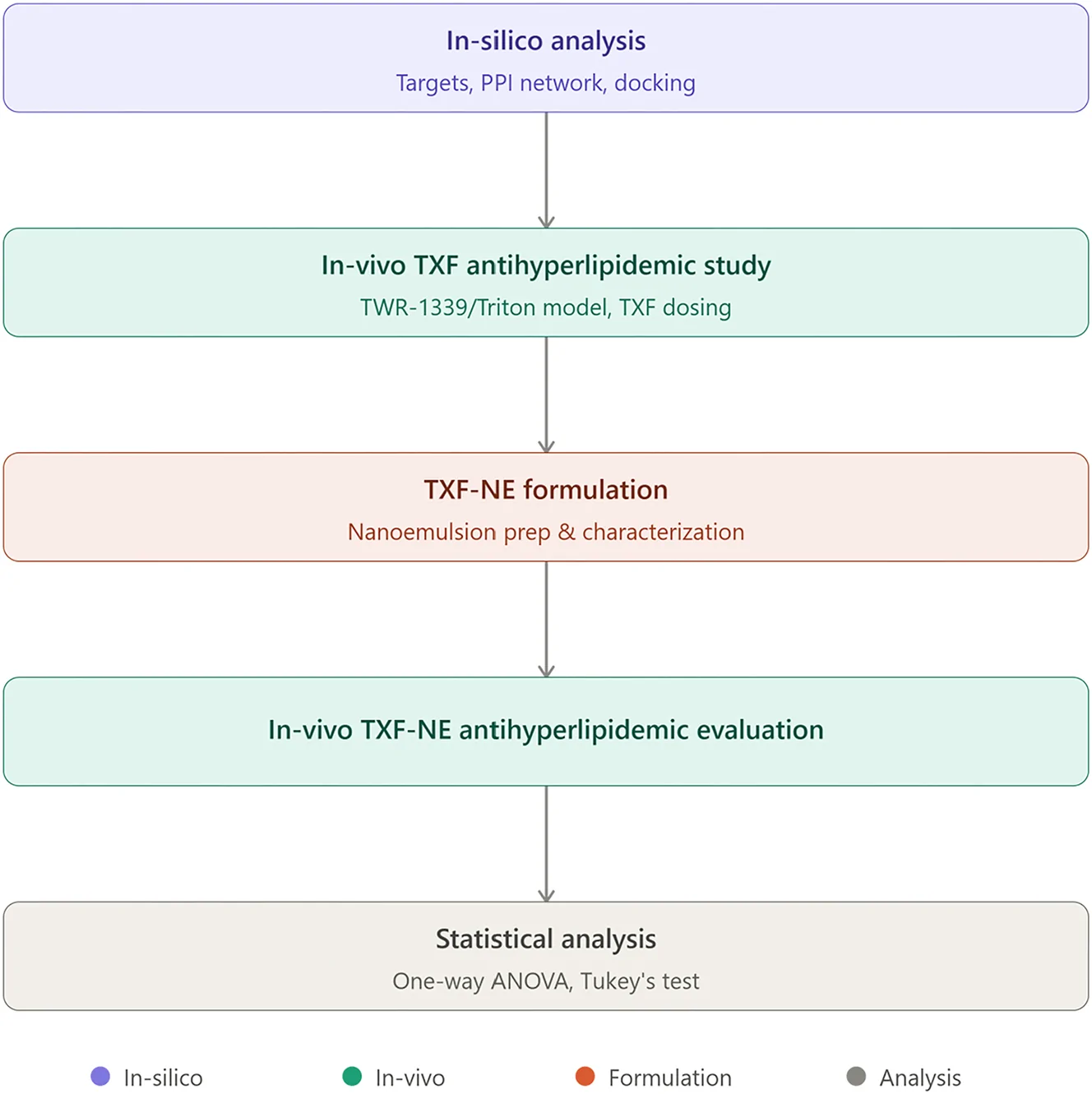
Flowchart of methods used for anti-hyperlipidemic evaluation of TXF.

Animals were divided into five groups with six animals in each group (*n* = 6).

1. Normal control: No treatment.
2. TWR-1339 (400 mg/kg): TWR-1339 single dose followed by triton X-100 for one week.
3. SIM (20 mg/kg): TWR-1339 single dose followed by triton X-100 and SIM for one week.
4. TXF-NE (50 mg/kg): TWR-1339 single dose followed by triton X-100 and TXF-NE (50 mg/kg) for one week.
5. TXF (50 mg/kg): TWR-1339 single dose followed by triton X-100 and TXF (50 mg/kg) for one week.

### 2.8 Statistical analysis

The data of biological replicates were expressed as mean ± standard deviation and were analyzed by One-way ANOVA followed by Tukey’s multiple comparison test using GraphPad Prism 8.02 software. A probability value of less than 0.05 was deemed as significant. The following acronyms were used to denote the level of significance, ***≤0.001, **≤0.01, *≤0.05.

## 3 Results

### 3.1 Identification of common targets for TXF and hyperlipidemia

The initial analysis involved retrieving potential targets for TXF using three different prediction platforms: PharmMapper (290), SuperPred (64), and WAY2DRUG (132). A total of 436 unique protein targets were identified. These targets were subsequently standardized through the UniProt database to obtain official gene symbols. Duplicates were removed ensuring that only distinct proteins were retained for further analysis. In parallel, a comprehensive search was conducted for hyperlipidemia-related targets using GeneCards (1384), DisGeNET (8), and the CTD (870). This search yielded 1,991 potential targets associated with hyperlipidemia. Following the consolidation of these results, the common targets between TXF and hyperlipidemia were identified. The Venn diagram illustrates the overlap between the targets for TXF and those associated with hyperlipidemia. Out of the total 2,427 targets (436 for TXF and 1,991 for hyperlipidemia), 177 targets were found to be common to both. The Venn diagram generated using Venny 2.1 represents these regions as 259 (11.5 %) for TXF, 177 (7.9 %) for overlapping targets, and 1814 (80.6 %) for hyperlipidemia. These values are internally consistent with the original datasets: 259 TXF-specific + 177 overlapping = 436 total TXF-related targets; 1,814 hyperlipidemia-specific + 177 overlapping = 1,991 total hyperlipidemia-associated targets. These common targets highlight the potential pathways through which TXF may exert its effects on hyperlipidemia, suggesting a shared mechanism of action (Figure 3).

**Figure 3:**
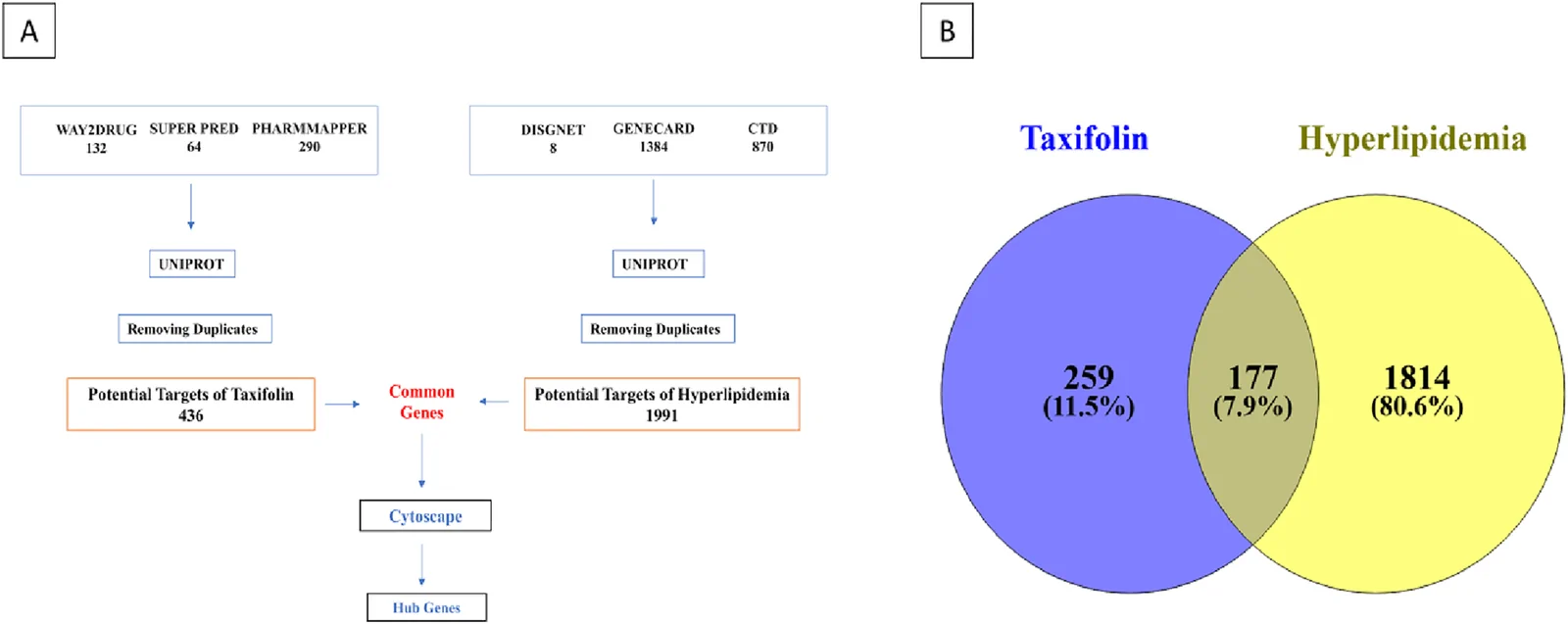
Identification of TXF’s targets associated with hyperlipidemia. (A) The flowchart details the process of target identification and consolidation through the UniProt database, (B) the Venn diagram shows that 177 targets overlap, indicating possible potential therapeutic intersections.

### 3.2 Bioinformatic prediction of hub genes and PPI network associated with the anti-hyperlipidemic effects of TXF

The PPI network analysis revealed a highly interconnected molecular framework comprising 176 nodes (proteins) and 2,410 edges (interactions), demonstrating a probable biological relevance in the context of TXF’s potential anti-hyperlipidemic effects. The network exhibited substantial enrichment (*p* < 1.0e-16) with an average node degree of 27.4, indicating that each protein interacted with approximately 27 others – a finding that far exceeds random expectation (986 edges) and suggests functionally coordinated pathways. The high average local clustering coefficient (0.544) further supports the presence of tightly interconnected protein modules, characteristic of key biological processes involved in lipid metabolism and inflammatory regulation. To identify highly connected nodes within the network, the cytoHubba plugin in Cytoscape was applied using the Degree ranking method. The top 50 hub genes were identified according to their degree scores (Figure 4; Table S2). The highest-ranked nodes were ALB (degree = 113), TNF (105), AKT1 (102), TP53 (93), ESR1 (88), CASP3 (83), BCL2 (83), PPARG (83), HSP90AA1 (79), and EGFR (78). Among the lipid-metabolism-related targets, PPAR-α ranked 15th (degree = 54), whereas HMGCR ranked 23rd (degree = 47). Other highly connected genes included MMP9, MAPK3, IGF1, GSK3B, CYP3A4, ACE, MMP2, MAPK1, STAT1 and others (Table S2). These results suggest that the common target network contains highly connected proteins that might be involved in lipid metabolism, inflammation, signaling, and cellular stress responses (Figure 4).

**Figure 4:**
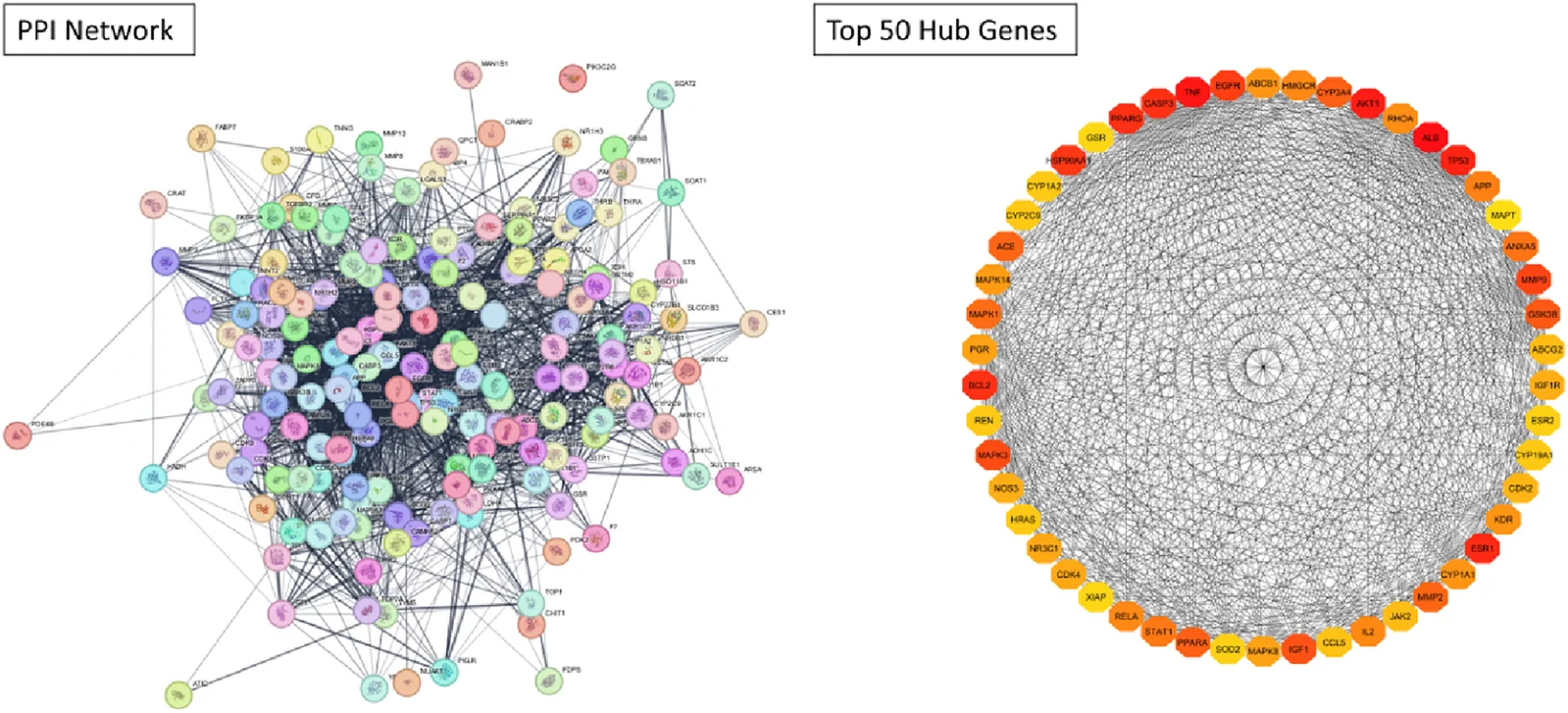
PPI network analysis of common TXF-hyperlipidemia targets. The network comprised 176 nodes and 2,410 edges. Hub genes were ranked using the Degree algorithm of the cytoHubba plugin in Cytoscape. The top 50 highly connected genes are represented in the network, with their degree scores provided in supplementary Table S1.

### 3.3 Gene ontology and pathway enrichment analysis predicted TXF’s multi-targeted action against hyperlipidemia

GO and KEGG enrichment analyses were performed to characterize the BPs and signaling pathways statistically associated with the common TXF–hyperlipidemia target set. In the BP category, the targets were significantly enriched in responses to xenobiotic stimuli, hormones, lipids, and oxygen-containing compounds, suggesting TXF’s role in modulating detoxification, metabolic regulation, and oxidative stress responses. Notably, terms like “monocarboxylic acid metabolic process” and “organic acid metabolic process” highlight its potential influence on fatty acid metabolism, while “cellular response to chemical stimulus” and “regulation of biological quality” imply broader homeostatic effects.

The MF analysis identified targets with nuclear receptor activity (e.g., ligand-activated transcription factors), oxidoreductase activity, and binding capacities for lipids, small molecules, and metal ions. This suggests that TXF could interact with transcriptional regulators (e.g., PPARs), enzymes involved in redox balance, and lipid transporters, which are critical for lipid metabolism and inflammation.

KEGG pathway enrichment revealed associations with atherosclerosis-related pathways (e.g., “Fluid shear stress and atherosclerosis,” “Lipid and atherosclerosis”), metabolic pathways (e.g., “FoxO signaling,” “PI3K-Akt signaling”), and stress responses (e.g., “HIF-1 signaling,” “AGE-RAGE signaling in diabetic comorbidities”). These align with TXF’s potential to mitigate lipid accumulation, oxidative stress, and inflammatory damage in hyperlipidemia. The high fold enrichment and low FDR values confirm the statistical robustness of these associations (Figure 5).

**Figure 5:**
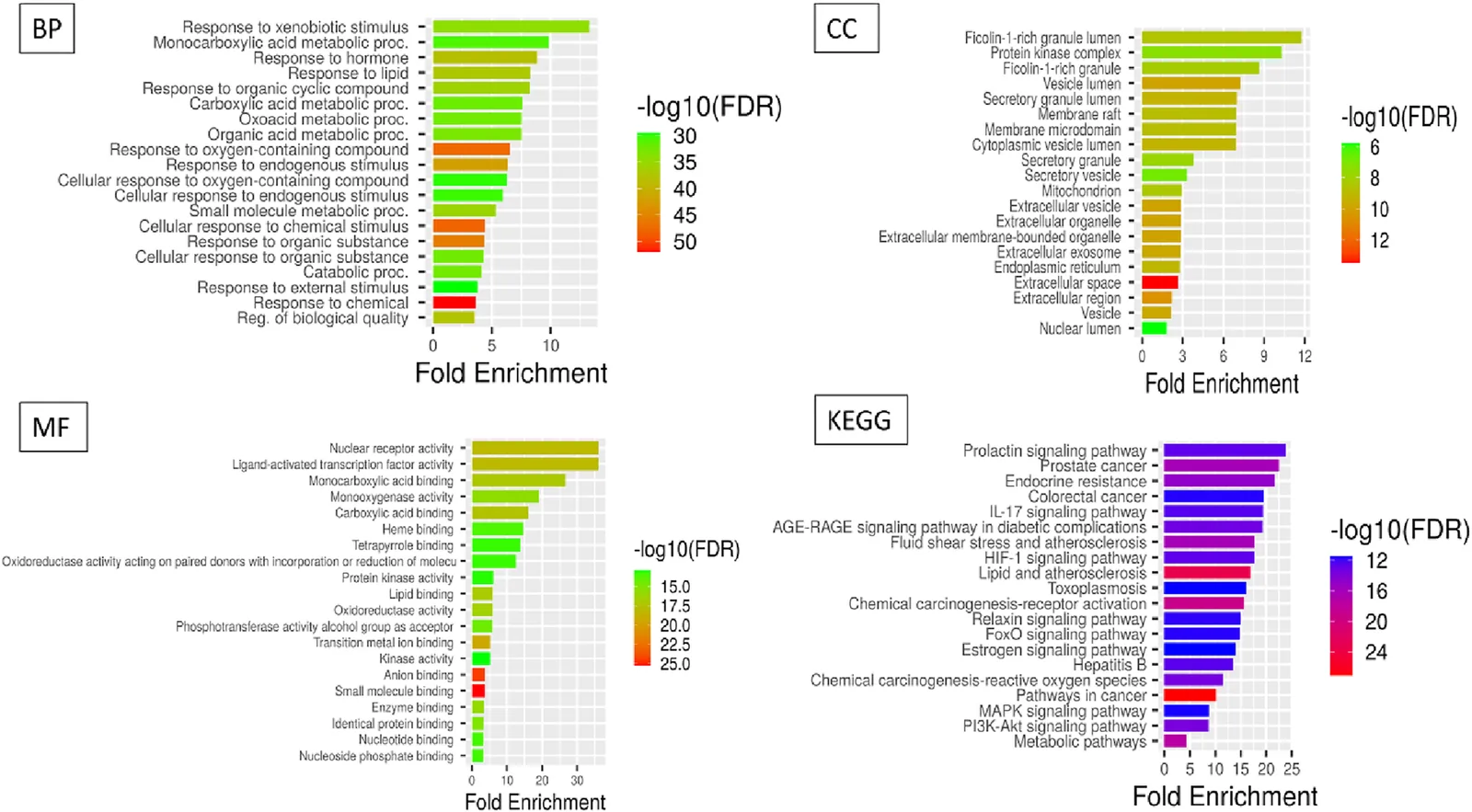
GO and KEGG enrichment analysis of the common TXF-hyperlipidemia target set. The enriched BPs and pathways are associated with lipid metabolism, cellular stress responses, inflammation, and related metabolic functions.

### 3.4 Molecular docking analysis reveals TXF’s binding interactions with key hyperlipidemia-related targets

Molecular docking analysis was performed to explore the potential structural interactions of TXF with five lipid-metabolism-related proteins, namely APOA1, HMGCR, LDLR, ACSM2A, and APOB, using the MOE Site Finder module to identify candidate binding regions, followed by Triangle Matcher placement, Induced Fit refinement, and London dG/GBVI-WSA dG scoring. HMGCR was considered for molecular docking because it was identified as a highly connected node in the PPI network and is a well-established regulator of cholesterol biosynthesis. LDLR and APOB were considered separately based on their established biological relevance to LDL clearance and atherogenic lipoprotein metabolism and their subsequent experimental evaluation in the present study. APOA1 and ACSM2A were additionally included as lipid-metabolism-related proteins for exploratory structural analysis based on their biological relevance. Thus, the molecular docking targets were selected using an integrated rationale combining network connectivity, biological relevance to hyperlipidemia, experimental findings, rather than solely on the basis of the top-50 hub ranking.

TXF showed favorable predicted docking scores across the investigated targets, ranging from −5.27 to −7.19 kcal/mol (Table 2). In the predicted APOA1 complex, TXF formed a hydrogen-bond interaction with GLU78 at approximately 2.63 Å. For HMGCR, TXF showed a predicted docking score of approximately −5.72 kcal/mol and formed a hydrogen-bond interaction involving GLU730 at approximately 2.9–2.97 Å, indicating a possible association with a region relevant to ligand recognition by HMGCR; however, this finding does not establish a direct enzymatic inhibition. In the predicted LDLR complex, TXF showed a π–H interaction with PRO640 at approximately 3.98 Å, while interactions involving LYS350 and VAL884 were observed in the predicted APOB complex at approximately 3.64–3.65 Å. TXF also adopted a favorable predicted pose within the identified binding region of ACSM2A. Overall, the docking models indicated that TXF can form different non-covalent interactions, including hydrogen bonds and π–H contacts, with the selected lipid-metabolism-related proteins. SIM generally showed more favorable docking scores than TXF; however, its comparison is most appropriately considered in the context of HMGCR, its established pharmacological target, rather than as a target-specific positive control for the other proteins. The predicted interactions provided a structural basis for further investigation of TXF protein associations (Table 2; Figure 6, S1).

**Table 2:** Docking analysis of TXF with various target proteins.

Compounds	S score (kcal/mol)	RMSD (Å)	Atom of compounds	Atom of receptors	Residue of receptor	Type of interactions	Distance (Å)	*E* (kcal/mol)
**APOA1 (PDB ID 1AV1)**								

TXF	−5.2711	1.0303	O18	OE1	GLU 78	H-donor	2.63	−4.2
SIM	−6.4565	2.1161	C24 O27	O OG1	TYR 192 THR 197	H-donor H-donor	3.32 2.68	−0.7 −0.9

**HMGCR (PDB ID 1HW8)**								

TXF	−5.7161	1.5413	O18 O18 O21 O21 O20 O20	OE1 OE1 OE1 OE1 N N	GLU 730 GLU 730 GLU 730 GLU 730 ALA 783 ALA 783	H-donor H-donor H-donor H-donor H-acceptor H-acceptor	2.97 2.97 2.9 2.9 3.14 3.14	−2 −2 −3.6 −3.6 −1.2 −1.2
SIM	−7.3100	1.3888	O29 O29 O29 O29	OE2 OE2 ND2 ND2	GLU 730 GLU 730 ASN 734 ASN 734	H-donor H-donor H-acceptor H-acceptor	2.9 2.9 3.1 3.1	−1.9 −1.9 −1 −1

**LDLR (PDB ID 1N7D)**								

TXF	−6.8937	1.3331	O18 O18 6-ring 6-ring	O O CD CD	VAL 592 VAL 592 PRO 640 PRO 640	H-donor H-donor pi-H pi-H	3.24 3.24 3.98 3.98	−2.1 −2.1 −0.6 −0.6
SIM	−7.4299	2.5240	O29 O29 O27 O27	O O N N	LEU 637 LEU 637 LEU 549 LEU 549	H-donor H-donor H-acceptor H-acceptor	2.96 2.96 2.94 2.94	−2.3 −2.3 −1.3 −1.3

**ACSM2A (PDB ID 2VZE)**								

TXF	−7.1864	1.1022	C7 C7 O20 O20 O10 O10	O O O O NE2 NE2	GLY 362 GLY 362 GLY 362 GLY 362 GLN 363 GLN 363	H-donor H-donor H-donor H-donor H-acceptor H-acceptor	3.21 3.21 2.85 2.85 3.47 3.47	−0.5 −0.5 −1.5 −1.5 −0.7 −0.7
SIM	−6.8071	2.0372	C25 C25 O29 O29 O29 O29 O7 O7	SD SD OE2 SD OE2 SD NH1 NH1	MET 383 MET 383 GLU 359 MET 383 GLU 359 MET 383 ARG 472 ARG 472	H-donor H-donor H-donor H-donor H-donor H-donor H-acceptor H-acceptor	3.7 3.7 2.78 3.41 2.78 3.41 3.34 3.34	−0.6 −0.6 −1.1 −0.7 −1.1 −0.7 −1.9 −1.9

**APOB (PDB ID 9EA7)**								

TXF	−6.7024	2.6217	O21 O21 6-ring 6-ring 6-ring 6-ring	ND2 ND2 CG CG2 CG CG2	ASN 346 ASN 346 LYS 350 VAL 884 LYS 350 VAL 884	H-acceptor H-acceptor pi-H pi-H pi-H pi-H	2.96 2.96 3.64 3.65 3.64 3.65	−0.8 −0.8 −0.5 −0.9 −0.5 −0.9
SIM	−8.7165	2.5672	O27 O27	ND2 ND2	ASN 886 ASN 886	H-acceptor H-acceptor	3.19 3.19	−1.2 −1.2

**Figure 6:**
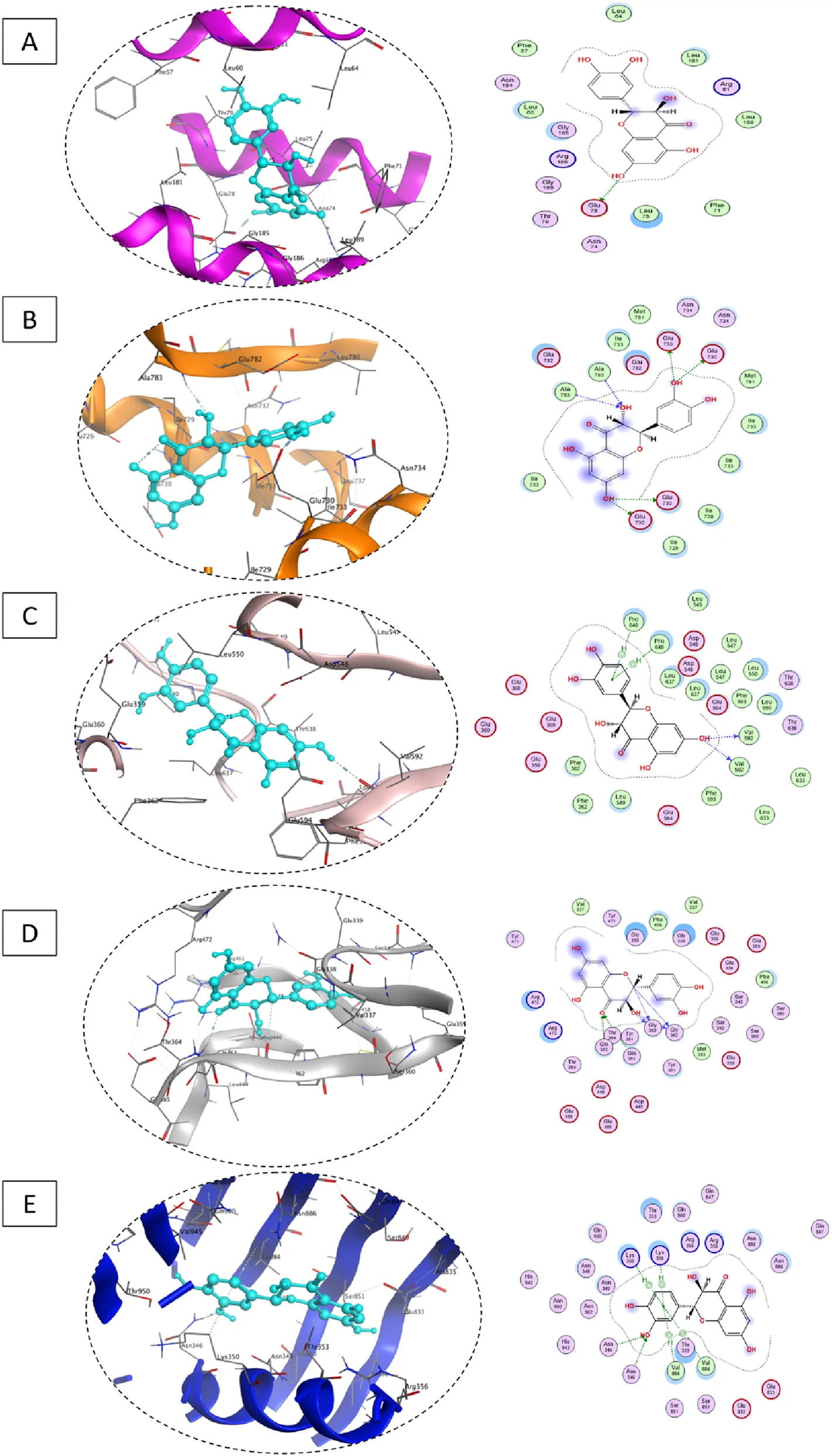
Molecular docking of TXF (Cyan) with hyperlipidemia-related targets. (A) APOA1 (PDB 1AV1), (B) HMGCR (PDB 1HW8), (C) LDLR (PDB 1N7D), (D) ACSM2A (PDB 2VZE) and (E) APOB (PDB 9EA7). The structures illustrate predicted hydrogen-bond and hydrophobic interactions between TXF and residues within the identified binding regions.

### 3.5 TXF attenuated the levels of TC, TG, LDL, VLDL and induced HDL levels

The levels of LDL in the treated groups (SIM 20 mg/kg, TXF 25 mg/kg, and TXF 50 mg/kg) were decreased compared to TWR-1339. In contrast to SIM and TXF treatment, which reduced LDL levels, the TWR-1339 group displayed higher LDL levels than the control group. TG levels in the TWR-1339 group were significantly higher than those in the control group. On the other hand, TXF (25 mg/kg, and 50 mg/kg) and SIM (20 mg/kg) significantly reduced TG levels and were equally effective in preventing TWR-1339-induced TG levels. Similar to LDL and TG, VLDL levels were elevated during the TWR-1339 regimen; however, the SIM and TXF groups (both doses) significantly decreased VLDL levels. TC level in the TWR-1339 group was somewhat above baseline, while the levels were much lower (***) for SIM and TXF treatments, indicating significant potential for cholesterol-lowering activities. Moreover, the HDL levels in the TWR-1339 group were lower than those in the control group. However, when SIM and TXF were administered, HDL levels were significantly raised; the greatest rise in HDL was seen when TXF was administered at 50 mg/kg (Figure 7).

**Figure 7:**
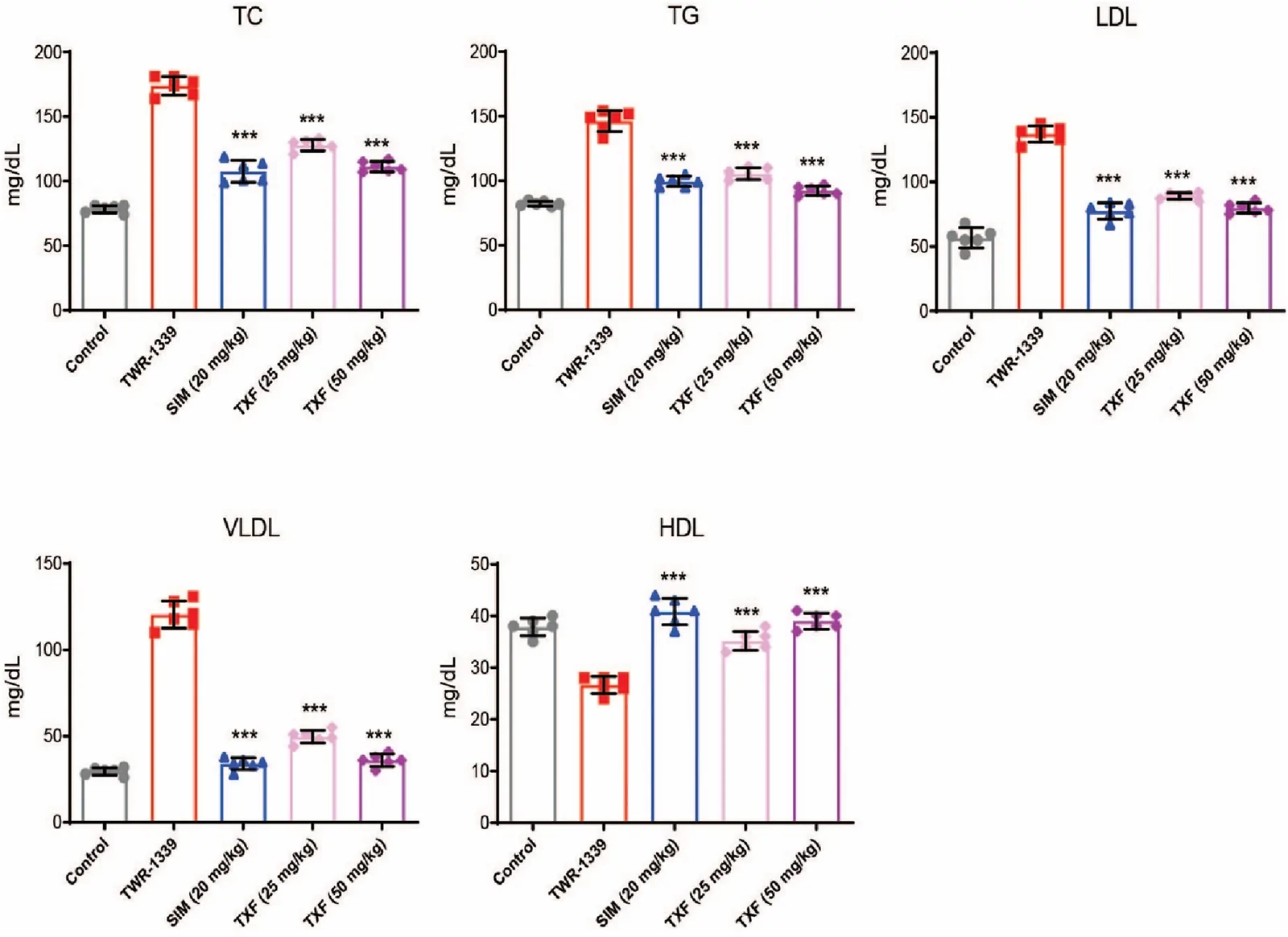
TXF reduced lipid levels in TWR-1339-induced hyperlipidemia. TWR-1339 induced highly noticeable hyperlipidemia, with low HDL and high levels of LDL, TG, and VLDL, as well as TC. These lipid alterations revert to normal levels with 50 mg/kg of TXF. *n* = 6; ***≤0.001 (treated vs. TWR-1339).

### 3.6 TXF attenuated the levels of LFTs in hyperlipidemic rats

LFT analysis demonstrated that treatment with TXF, particularly at the 50 mg/kg, significantly reduced AST and ALP levels compared to the SIM (20 mg/kg) group, indicating a potential hepatoprotective effect. SIM treatment led to elevated levels of these enzymes, suggestive of liver stress or damage, while TXF administration mitigated these increases, especially in the ALP profile where both TXF doses showed a marked reduction. ALT levels remained relatively stable across all groups, implying limited hepatocellular injury. These findings suggest that unlike statins, TXF can protect against hepatic dysfunction associated with hyperlipidemia (Figure S2).

### 3.7 TXF reduced the transcript and protein levels of HMGCR, APOB and induced LDLR expression

All treatment groups showed significant decrease in HMGCR expression compared to the TWR-1339 group. The expression levels gradually declined as TXF dosages increased, indicating a dose-dependent effect. The APOB expression profile displays different patterns within the same treatment groups. Compared to the control, all treatment groups exhibited noticeably reduced expression levels. The results highlight the therapy’s ability to control APOB by showing a consistent and statistically significant suppression of this parameter. The therapies significantly upregulated LDLR compared to the control. In the treated groups, there was a statistically significant increase in LDLR expression; stronger responses were obtained with a larger TXF dose. These findings indicate that TXF inhibits TWR-1339-induced hyperlipidemia via differential regulation of lipid-modulating targets. Together, these observations highlight the TXF’s therapeutic potential in controlling receptor-mediated clearance, lipid transport, and cholesterol production (Figure 8).

**Figure 8:**
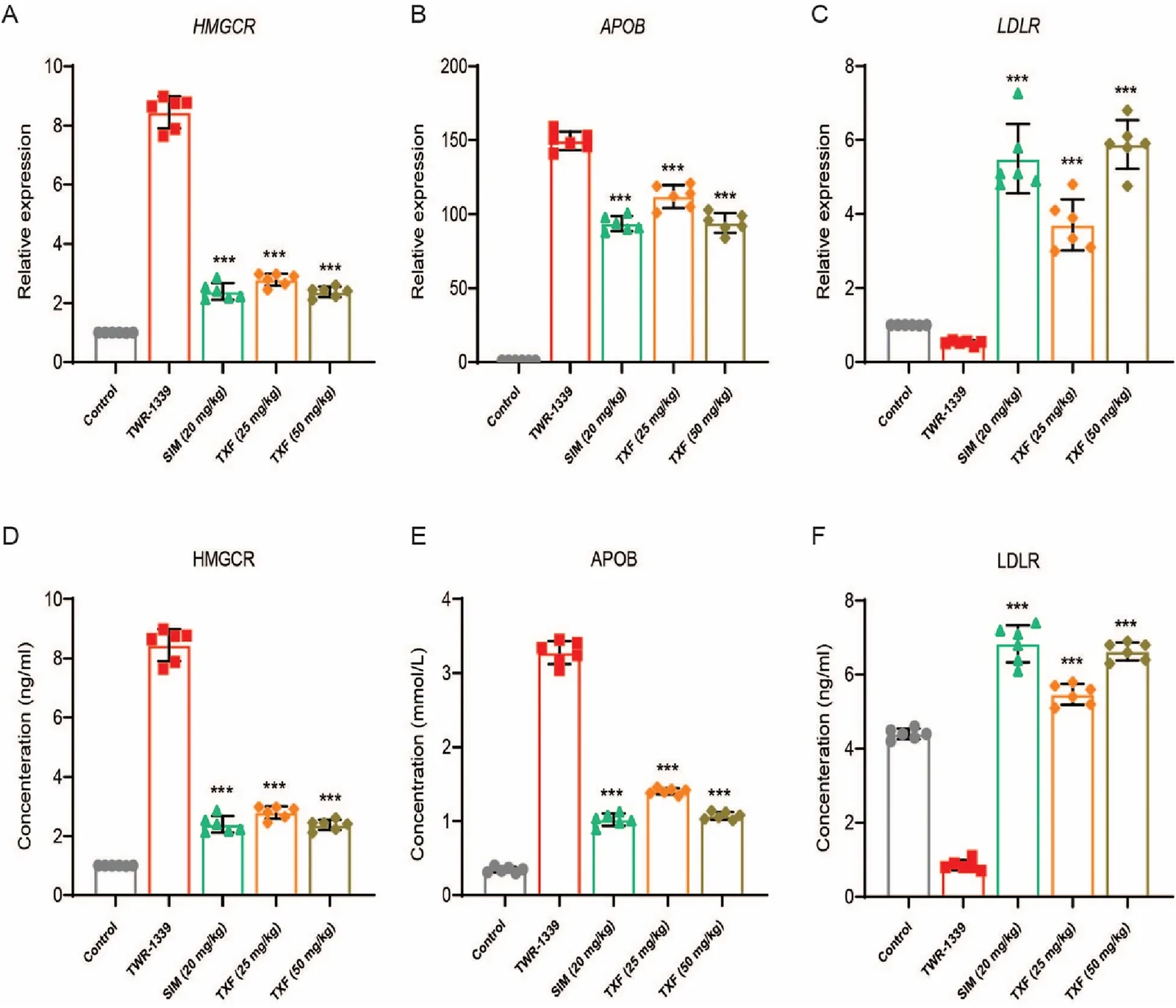
TXF attenuated the transcript and protein levels of HMGCR and APOB while upregulating LDLR expression. (A), (B), (C) Transcript levels, (D), (E), (F) protein concentration. *n* = 6; ***≤0.001 (treated vs. TWR-1339).

### 3.8 Visual appearance assessment

The prepared formulation was visually examined to assess its physical appearance immediately after preparation. No phase separation, precipitation or visible aggregation was observed and the formulation remained homogeneous, clear and transparent, suggesting adequate solubilization and mixing of the formulation components at this initial time point.

### 3.9 Zeta potential and particle size measurement supported TXF’s nanoscale attributes of formulation

TXF-NE exhibited mean diameter, polydispersity index and zeta potential of 239 ± 29.6, 0.15 ± 0.09 and −45.1 ± 6.11 (Mean ± SD) respectively. The nanoscale droplet size falls within the desirable range for NE-based delivery systems, expected to enhance the aqueous solubility and permeability of the poorly water-soluble flavonoid TXF. The low PDI value indicates a homogeneous and narrow droplet size distribution, suggesting uniform emulsification and a physically stable colloidal system. The zeta potential of −45.1 mV, exceeding the ±30 mV threshold generally associated with good colloidal stability, reflects strong electrostatic repulsion between droplets, which is expected to minimize aggregation and coalescence during storage (Figure 9).

**Figure 9:**
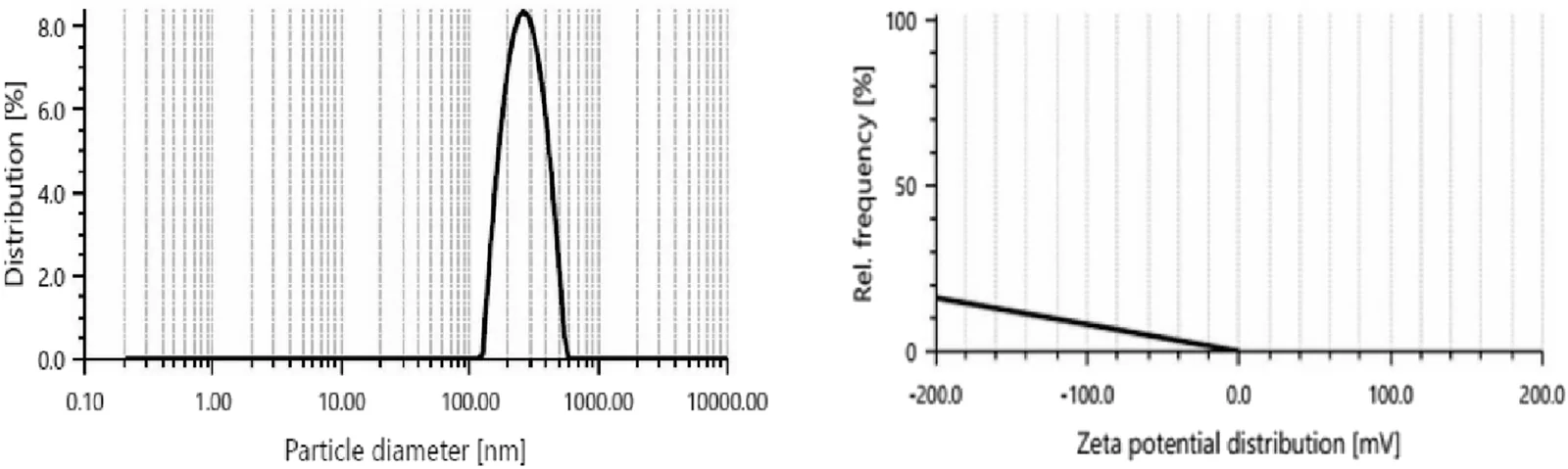
Particle-size and zeta-potential of TXF-NE.

### 3.10 Drug content estimation in formulation

The drug content of TXF-NE was determined using UV-visible spectrophotometry at a maximum absorption wavelength (*λ*_max_) of 289 nm. The absorbance values of the sample solutions were compared with the absorbance of standard (TXF), and the drug content was calculated accordingly. The drug content in the TXF-NE samples ranged from 89.1 % to 91.9 %, indicating a high drug content (Table 3). The high drug content suggests that the formulation approach, including the selection of excipients, emulsification method, and process parameters, was optimized to ensure minimal drug loss. However, further studies would be required to confirm whether the drug content remains stable, preventing potential drug degradation or precipitation.

**Table 3:** Drug content determination in TXF-NE.

Sr. No	Λmax	Absorbance of sample	Absorbance of standard	Drug content
1	289 nm	0.573	0.623	91.9
2	289 nm	0.551	0.617	89.1
3	289 nm	0.561	0.622	90.1

### 3.11 Characterization of TXF-NE

The FTIR spectrum of PEG 400 presented a characteristic peak at 3,400 cm^−1^, and 2,800-2,900 cm^−1^ that belongs to the O-H and C-H stretch respectively. The other peaks that were prominent appeared at 1,670 cm^−1^ and 1,450 cm^−1^ presenting the presence of a carbonyl group and C-H bend in PEG 400. Peppermint oil is composed of different components, containing menthol, menthone, limonene, and beta-pinene. The FTIR spectrum presented a characteristic peak of the O-H stretch, and C-H stretch at 3,500 cm^−1^ and 2,800-2,900 cm^−1^ respectively. The other peaks that were witnessed in the FTIR spectrum of peppermint oil include peaks of ester linkage at 1740 cm^−1^ whereas peaks appearing at 1,470 cm^−1^ and 1,100 cm^−1^ belong to the C-H bend and C-O stretch respectively. In the FTIR spectrum of tween 80, the characteristic peak at 3,300-3,500 cm^−1^ indicates the presence of a hydroxyl group within tween 80 whereas the peak appearing at 2,800-2,900 cm^−1^ represents the C-H stretch. The other peaks at 1713 cm^−1^, 1,458 cm^−1,^ and 1,040 cm^−1^ present the carbonyl group, C-H bend, and C-O stretch respectively.

The FTIR spectrum of TXF-NE exhibited a characteristic peak at 3,400 cm^−1^ and 2,800-2,900 cm^−1^ which indicates the presence of a hydroxyl group and C-H stretch in the TXF. The other peaks apparent at 1,670 cm^−1^ is indicative of the carbonyl group, whereas peaks appearing at 1,400 cm^−1^ represent the C-C stretch in the TXF. In the FTIR spectrum of emulsion, the representative peak of all the individual components indicates the incorporation of active pharmaceutical ingredient into the emulsion (Figure S3).

### 3.12 NE-based delivery potentiates the lipid-lowering effects of TXF

Administration of TWR-1339 markedly disrupted the lipid profile, as evidenced by significant elevations in TC, TG, LDL, and VLDL levels along with a reduction in HDL compared with the control group. Treatment with TXF-NE (50 mg/kg) substantially ameliorated these alterations, producing a greater reduction in TC, TG, LDL and VLDL levels than TXF (TXF, 50 mg/kg) and yielding values that were comparable to or better than those observed with SIM. Notably, TXF-NE exhibited the most pronounced improvement in LDL and VLDL concentrations while also producing the highest increase in HDL levels among the treatment groups. These findings indicate that NE-based delivery enhances the anti-hyperlipidemic efficacy of TXF, an effect that may plausibly involve improved bioavailability, although this was not directly assessed in the present study.

Assessment of LFTs further demonstrated the better effects of TXF-NE. Compared with TXF, the NE-formulation produced a greater reduction in serum AST, ALT and ALP activities, suggesting attenuation of hepatic injury associated with dyslipidemia. TXF-NE consistently showed a stronger restorative effect, bringing enzyme values closer to those of the control animals. Collectively, these results suggest that NE-based delivery significantly enhances both the lipid-lowering and hepatoprotective properties of TXF, supporting its potential as an effective therapeutic strategy for the management of hyperlipidemia and associated hepatic dysfunction (Figure 10).

**Figure 10:**
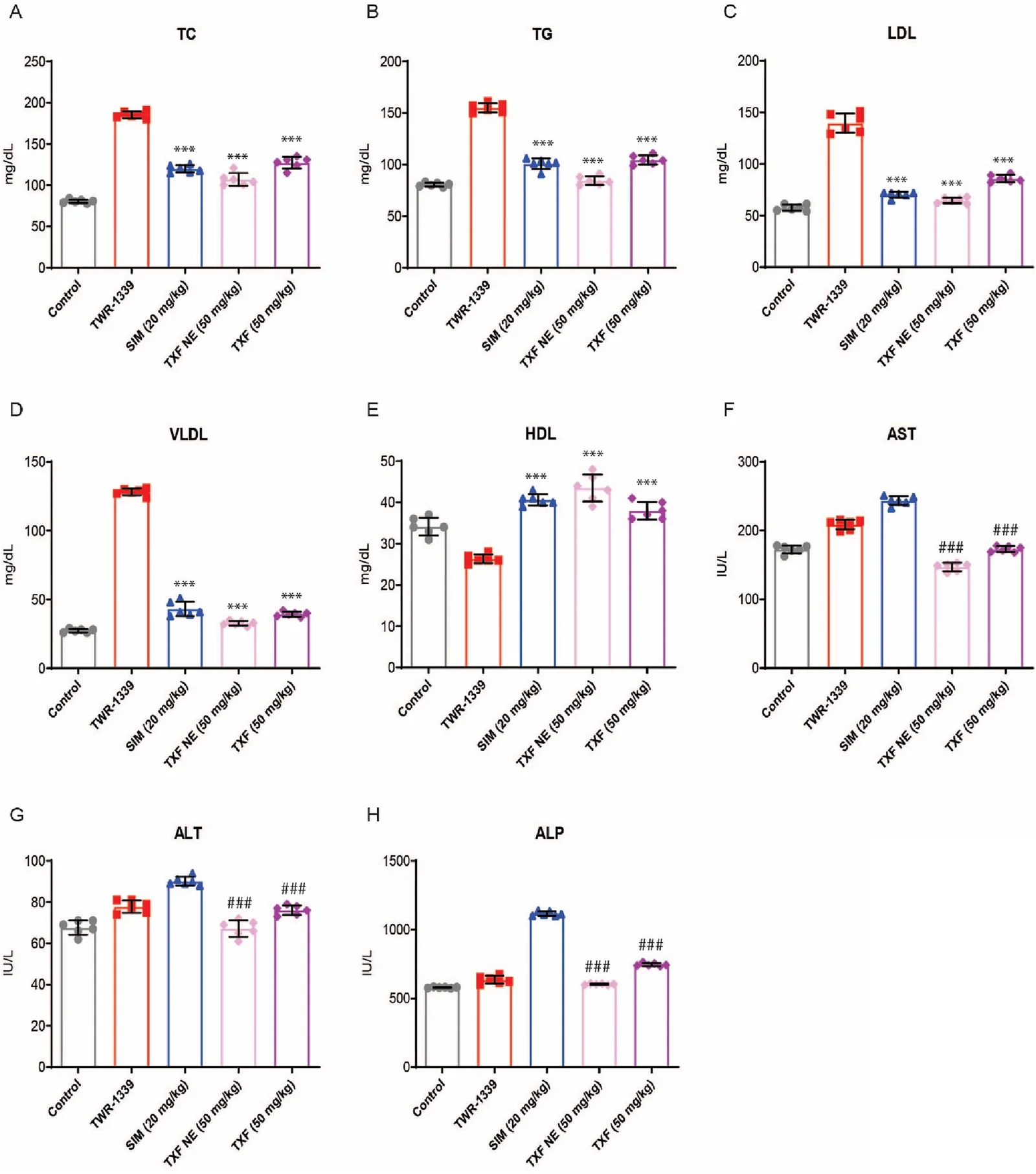
TXF-NE improved lipid profile and liver enzyme levels in hyperlipidemic rats. TXF-NE displayed better anti-hyperlipidemic when compared to TXF. *n* = 6; ***≤0.001 (treated vs. TWR-1339), ###≤0.001 (treated vs. SIM).

## 4 Discussion

Natural plant-derived bioactive compounds have emerged as promising candidates due to their ability to modulate multiple molecular targets and signaling pathways involved in lipid metabolism. Their favorable safety profiles and broad-spectrum pharmacological activities further support their potential in the prevention and management of dyslipidemia and associated cardiovascular complications [58].

In the present study, the anti-hyperlipidemic potential of TXF and its NE-formulation was evaluated in a TWR-1339-induced hyperlipidemia model. The findings demonstrated significant lipid-lowering effects of TXF, highlighting its therapeutic potential against hyperlipidemia. TXF is a naturally occurring flavonoid that has previously been reported to possess diverse pharmacological properties, including antioxidant, anti-inflammatory, anticancer, and cardioprotective activities. Compared with several other flavonoids, such as quercetin, TXF exhibits lower toxicity and lacks mutagenic effects, thereby providing a favorable safety profile [59], 60]. The absence of observable toxicity or behavioral changes in the experimental animals further supports its potential for therapeutic use in hyperlipidemia management [60].

Dysregulation of hepatic cholesterol metabolism remains a hallmark of hyperlipidemia and plays a pivotal role in the development of atherosclerosis and CVDs. Therefore, targeting key regulators of cholesterol synthesis and transport represents an important therapeutic strategy for controlling lipid disorders [61]. One of the most notable findings of the present study was the ability of TXF to modulate the expression of HMGCR, the rate-limiting enzyme in cholesterol biosynthesis. HMGCR catalyzes the conversion of HMG-CoA to mevalonate, a critical step in the mevalonate pathway responsible for endogenous cholesterol production [62]. Increased HMGCR activity promotes excessive cholesterol synthesis and contributes to the progression of hyperlipidemia [63]. Our results demonstrated that TXF significantly attenuated HMGCR transcript levels in hyperlipidemic animals, suggesting suppression of hepatic cholesterol biosynthesis. This effect resembles the pharmacological action of statins and is consistent with previous studies highlighting the importance of HMGCR inhibition in lowering LDL cholesterol levels and reducing cardiovascular risk [64], 65].

In addition to inhibiting cholesterol synthesis, TXF influenced the expression of hepatic LDLR, a key mediator of cholesterol homeostasis. LDLR facilitates the hepatic uptake and clearance of circulating LDL particles, thereby reducing plasma LDL cholesterol levels. The expression of LDLR is tightly regulated by sterol regulatory element-binding proteins (SREBPs), particularly SREBP-1, which plays an important role in lipid synthesis and accumulation [66], 67]. The present study revealed a significant upregulation of hepatic LDLR expression following TXF treatment, indicating enhanced hepatic clearance of LDL cholesterol from the circulation. Increased LDLR expression is widely recognized as an effective mechanism for improving lipid profiles [66] and reducing cardiovascular risk, further supporting the anti-hyperlipidemic activity of TXF.

Another important molecular target affected by TXF was APOB, a structural protein essential for the assembly, secretion, and transport of atherogenic lipoproteins, including VLDL, LDL and chylomicrons. Elevated APOB levels are strongly associated with increased concentrations of circulating atherogenic lipoproteins and a higher risk of atherosclerosis and CVDs [68]. Accordingly, inhibition of APOB synthesis has emerged as a promising therapeutic approach for the treatment of dyslipidemia, with both genetic and pharmacological studies demonstrating significant reductions in LDL cholesterol following APOB suppression [69], [70], [71]. In the present study, TXF markedly reduced APOB transcript levels, suggesting a decrease in the production and circulation of atherogenic lipoproteins. These findings are in agreement with previous reports indicating that APOB downregulation contributes to improved lipid homeostasis and reduced plasma LDL concentrations [72], 73]. Collectively, the findings of this study indicate that TXF exerts its anti-hyperlipidemic effects through a multifactorial mechanism involving the suppression of cholesterol biosynthesis, enhancement of LDL clearance, and reduction of atherogenic lipoprotein production. The coordinated downregulation of HMGCR and APOB, together with the upregulation of LDLR, highlights the ability of TXF to target multiple key pathways involved in lipid metabolism.

Though pharmacokinetic and tissue-distribution studies were not performed in this study, the enhanced efficacy of TXF-NE may be discussed in light of well-established properties of NE-based delivery systems. The enhanced therapeutic performance of TXF-NE may be attributed to the inherent advantages of NE-based delivery systems, including improved aqueous solubility, enhanced gastrointestinal absorption, increased bioavailability, and prolonged systemic circulation of poorly water-soluble phytochemicals. NEs also facilitate efficient cellular uptake and tissue distribution owing to their nanoscale droplet size and large surface area, thereby increasing the amount of active compound reaching the target tissues [74]. These properties are generally associated with NE-based systems and may plausibly contribute to the stronger downregulation of HMGCR and APOB and the enhanced upregulation of LDLR. Therefore, NE-based delivery appears to be an effective strategy for maximizing the therapeutic potential of TXF and may represent a promising approach for improving the clinical applicability of flavonoid-based interventions in hyperlipidemia. It should be noted, however, that this study did not include a blank nanoemulsion (excipient-only) control group. As peppermint oil, Tween 80, and PEG 400 have themselves been reported to exert mild biological effects in some contexts, a possible contribution of the excipients to the observed effects cannot be entirely excluded.

## 5 Conclusions

These findings highlight TXF as a promising candidate for hyperlipidemia treatment, with a mechanism of action that aligns with established lipid-lowering strategies. The study’s results provide a foundation for further exploration of TXF’s therapeutic potential in clinical settings. Future studies should focus on long-term safety assessments, dose optimization, tissue distribution evaluations, inclusion of a blank-NE control to isolate excipient effects and potential combination therapies to enhance its efficacy. Additionally, clinical trials will be necessary to determine its effectiveness in the human population and establish its role in the management of cardiovascular diseases.

## Supplementary Material

Supplementary Material

Supplementary Material
