# Supplementary material for: Taxifolin targets HMGCR, LDLR and APOB to ameliorate hyperlipidemia: insights from systems pharmacology and nanoemulsion-based delivery
Source: Open Life Sci. 2026 Oct 22;21(1):20251392. doi: 10.1515/biol-2025-1392 (PMC13686381; doi:10.1515/biol-2025-1392)
Supplement: Supplementary file 1 [file j_biol-2025-1392_suppl_002.docx]

**Table S1: Primers used for RT-qPCR analysis**

| Sr.  No. | Gene | Primers | Sequences (5’ - 3’) | BP |
| --- | --- | --- | --- | --- |
| 1 | LDLR | LDLR F: | GGGTTCCATAGGGTTTCTGCT | 21 |
|  |  | LDLR R: | TGGTATACTCGCTGCGGTCC | 20 |
| 2 | HPRT | HPRT F: | CTCATGGACTGATTATGGACAGGAC | 25 |
|  |  | HPRT R: | GCAGGTCAGCAAAGAACTTATAGCC | 25 |
| 3 | APOB | ApoB F: | GTAGTGGTGCGTCTTGGATC | 20 |
|  |  | ApoB R: | TGCAGCAAGCTGTTGAATGT | 20 |
| 4 | HMGCR | HMGCR F: | CCTCCATTGAGATCCGGAGG | 20 |
|  |  | HMGCR R: | AAGTGTCACCGTTCCCACAA | 20 |

**Table S2: Top 50 hub genes along with their respective degree Scores.**

| **Rank** | **Name** | **Score (Degree Method)** |
| --- | --- | --- |
| 1 | ALB | 113 |
| 2 | TNF | 105 |
| 3 | AKT1 | 102 |
| 4 | TP53 | 93 |
| 5 | ESR1 | 88 |
| 6 | CASP3 | 83 |
| 6 | BCL2 | 83 |
| 6 | PPARG | 83 |
| 9 | HSP90AA1 | 79 |
| 10 | EGFR | 78 |
| 11 | MMP9 | 72 |
| 12 | MAPK3 | 70 |
| 13 | IGF1 | 61 |
| 14 | GSK3B | 58 |
| 15 | PPARA | 54 |
| 15 | CYP3A4 | 54 |
| 17 | ACE | 53 |
| 17 | MMP2 | 53 |
| 19 | MAPK1 | 52 |
| 20 | ANXA5 | 51 |
| 21 | STAT1 | 49 |
| 22 | APP | 48 |
| 23 | HMGCR | 47 |
| 23 | IL2 | 47 |
| 23 | RELA | 47 |
| 26 | RHOA | 45 |
| 27 | CYP1A1 | 44 |
| 27 | ABCB1 | 44 |
| 27 | KDR | 44 |
| 30 | MAPK14 | 43 |
| 31 | MAPK8 | 42 |
| 31 | PGR | 42 |
| 33 | CDK4 | 41 |
| 33 | NR3C1 | 41 |
| 35 | NOS3 | 40 |
| 35 | IGF1R | 40 |
| 37 | CDK2 | 39 |
| 38 | JAK2 | 38 |
| 38 | ABCG2 | 38 |
| 40 | CCL5 | 37 |
| 41 | CYP1A2 | 36 |
| 41 | CYP19A1 | 36 |
| 41 | REN | 36 |
| 41 | HRAS | 36 |
| 41 | CYP2C9 | 36 |
| 46 | ESR2 | 35 |
| 46 | SOD2 | 35 |
| 48 | GSR | 33 |
| 48 | XIAP | 33 |
| 50 | RXRA | 32 |

**Figure S1: Comparative molecular docking of SIM (Yellow) with hyperlipidemia targets.** (A) APOA1 (PDB 1AV1), (B) HMGCR (PDB 1HW8), (C) LDLR (PDB 1N7D), (D) CoA-Synthase (PDB 2VZE), and (E) APOB (PDB 9EA7) demonstrate SIM's binding interactions (hydrogen bonds and hydrophobic contacts) with key residues, highlighting its stronger affinity with lipid-regulating proteins.

**Figure S2: Hepatoprotective effects of TXF.** TXF at 50 mg/kg decreased liver enzyme levels. n = 6; *** ≤ 0.001, ** ≤ 0.01, * ≤ 0.05.

**Figure S3: FTIR spectra of PEG 400, TXF-NE, peppermint oil, tween 80, and TXF**
